# “If you will counsel properly with love, they will listen”: A qualitative analysis of leprosy affected patients’ educational needs and caregiver perceptions in Nepal

**DOI:** 10.1371/journal.pone.0210955

**Published:** 2019-02-06

**Authors:** Jorge César Correia, Alain Golay, Sarah Lachat, Suman Bahadur Singh, Varsha Manandhar, Nilambar Jha, François Chappuis, David Beran, on behalf of the COHESION Project

**Affiliations:** 1 Division of Tropical and Humanitarian Medicine, Geneva University Hospitals and University of Geneva, Geneva, Switzerland; 2 Division of Therapeutic Patient Education for Chronic Diseases, Geneva University Hospitals, Geneva, Switzerland; 3 School of Public Health and Community Medicine, B. P. Koirala Institute of Health Sciences, Dharan, Nepal; Imperial College London, UNITED KINGDOM

## Abstract

**Background:**

Leprosy remains a disease of concern in many countries including Nepal. To achieve the target of elimination, the WHO strategy promotes comprehensive education of patients, healthcare workers (HCWs), and the public on leprosy-related issues. However most educational programs are based on the concerns of HCWs and not on patients’ needs. The objective of this paper is to explore the educational needs of leprosy affected patients in Nepal and compare them to the needs perceived by HCWs.

**Methodology/principal findings:**

Semi directive interviews were conducted with patients and HCWs. The data was analyzed using the basic interpretative qualitative framework. The study was conducted in two leprosy referral centers, one university hospital and one primary health care center: Lalgadh Leprosy Hospital and Services Centre, Anandaban Hospital and its satellite clinic in Patan, B. P. Koirala Institute of Health Sciences in Dharan, and the Itahari primary health care centre.

The results show that there remains a lack of knowledge regarding the disease (origins, manifestations, prevention and treatment) contributing to late care seeking behavior and high levels of stigma, with an important psychological and financial stress for patients. All of the HCWs displayed a good understanding of patients’ difficulties and needs and acknowledged the key role of patient education. However, they expressed several challenges in managing patients due to lack of time, human resources and training in patient education.

**Conclusions/significance:**

Further efforts need to be made to increase patients’ general knowledge of the disease in order to motivate them to seek healthcare earlier and change their perception of the disease to reduce stigma. HCWs need proper training in patient education and counseling for them to acquire the necessary skills required to address the different educational needs of their patients. The use of lay and peer counselors would be an option to address the workload and lack of time expressed by HCWs.

## Introduction

Leprosy remains a disease of concern in many countries of Africa, southeast Asia, and the Americas where more than 200 000 new cases, including around 25 000 infections in children, are being diagnosed every year [1,2]. In 1991, the 44th World Health Assembly adopted the target of elimination of leprosy as a public health problem, defined as reducing the prevalence to less than 1 case per 10,000 population by the year 2000 [3]. Today, of the 122 countries in which leprosy is still endemic, 120 have reached the WHO elimination goal, mainly thanks to the widespread availability of multidrug therapy (MDT) [4].

Although this target has been met at a global level and in many cases at a national level, elimination of leprosy has not been achieved at a subnational level [5]. This is the case in Nepal, where leprosy was eliminated at the national level in 2009, and declared so in 2010 with the registered prevalence rate of 0.77 case per 10,000 population. However, several districts still have a prevalence above the elimination threshold [6]. At the end of the fiscal year 2015/16, the number of districts in Nepal reporting a prevalence rate of more than 1 per 10,000 population increased to 18 from 15 in the previous year out of a total of 75 districts [7].

In 2016, WHO published the Global Leprosy Strategy 2016–20 [8], which includes as one of its main strategies the comprehensive education of patients, healthcare workers (HCWs), and the public on leprosy-related issues. Patient education is an integral part of leprosy control, but its role in leprosy disease control is often underrated and misunderstood [9]. The International Federation of Anti-Leprosy Associations (ILEP) states that leprosy education should produce behavioral change in overcoming the sociocultural aspects of leprosy stigma, identification and recognition of the disease, continuation of anti-leprosy therapy, and promotion of rehabilitation (both social and physical) [10]. There is increasing evidence that patient education and counseling for leprosy results in increased knowledge, change of behavior and a reduction of stigma [11–15].

The design of educational programs and their implementation are most often based on the concerns or interests of HCWs. However, the HCW is aware of a number of educational needs not perceived by the patient, conversely, the patient is aware of a number of needs related to his daily life not perceived by the HCWs [16–19]. Educational needs refer to the gap between the current skills the patient possesses and skills that need to be acquired that will allow the individual to better manage their illness and treatment [20]. Developing an educational program based on the patients’ needs allows for a patient-centered approach, which has proven to improve patients health status and increase the efficiency of care [21–23].

Therefore, this study explores the educational needs of leprosy affected patients in Nepal and compares them with the needs identified by HCWs. The aim was to contribute to a reflection on educational activities needed to be developed or reinforced by the actors involved in the care of leprosy affected patients not only in Nepal, but worldwide.

## Methodology

### Data collection and analysis

Findings from this research were obtained during fieldwork in Nepal conducted from January 31^st^ to February 10^th^, 2017. The questionnaires were developed by JC and AG based on the researchers’ experience on patient education and further adapted and validated by SS and VM experienced in the care of leprosy affected patients in Nepal. JC and SL conducted semi directive interviews, using two sets of open-ended questionnaires, one for patients (S1 File) and one for HCWs (S2 File) with SS translating.

Topics addressed in the interviews were the “5 dimensions of the educational diagnosis” as described by d’Ivernois and Gagnayre [24]. This is a widely used framework in the field of therapeutic patient education, allowing a systematic and detailed collection of information that can serve as a basis for building a personalized education program [25–27]. The dimensions in question are: biomedical (what does he/she have?), socio-professional (what does he/she do?), cognitive (what does he/she know about his illness?), psycho-affective (who is he/she?) and the persons projects (what are his/her projects?).

To analyze the data we utilized the basic interpretative qualitative framework [28]. The participants’ narration and statements were recorded using a digital audio recorder. Each interview was replayed from the recordings and transcribed (converted to text). JC and SL independently selected sections from the interviews relevant to the research objectives and coded these into keywords and themes. Both coders discussed the thematic analysis of responses and deviant cases, compared interpretations, and reached agreement on recurring themes. Finally, coded themes were organized into the 5 dimensions of the educational diagnosis according to the model developed by d’Ivernois and Gagnayre [24].A matrix was used to organize specific statements from patients and HCWs which allowed a comparison. The investigators held interviews until saturation was achieved, i.e. when subsequent interviews did not produce any new information. The researchers agreed when thematic saturation was achieved. The consolidated criteria for reporting qualitative research (COREQ) were followed [29].

### Research participants

A heterogeneous, purposive sampling approach [30] was used when recruiting the patients and HCWs during the visits of the different selected sites. The inclusion criteria for patients were (a) clinically diagnosed with leprosy, (b) willingness and psychological readiness to participate and c) able to communicate in English or a Nepali dialect spoken by the translator (SS). As for the HCWs, all those involved in the care of leprosy affected patients and willing to participate in the designated healthcare facilities were selected. Sampling was determined using data saturation principles, with continual sampling until new data collected did not provide any new insights.

### Study sites

The study was conducted in four hospitals in Nepal including two leprosy referral hospitals, Lalgadh Leprosy Hospital and Services Centre (LLHSC) and Anandaban Hospital and its satellite clinic in Patan, one general referral hospital (B. P. Koirala Institute of Health Sciences (BPKIHS) in Dharan) and one primary health care centre (PHC) in Itahari. These sites were selected for the large number of leprosy patients they attend to, being representative of different levels of the health system and being located in different regions of Nepal. The aim was to get a broad picture of leprosy care and educational practices in Nepal through this sampling approach.

### Ethics

This research received ethical approval from Nepal and Switzerland (Nepal Health Research Council-NHRC, Commission cantonale d’éthique de la recherche de Genève) as part of a larger project (www.cohesionproject.info). We obtained oral consent by all the participants before each interview. For participants under age 18 (n = 1), consent was obtained from their parents who were present during the interview. Oral consent was chosen over written consent in order to avoid problems related to low literacy levels. The ethics committee approved the verbal consent procedure. The purpose and objectives of the study and introductions by the researchers were done prior to data collection. Permission on utilizing aids for recording the interview process was also presented to the participants and all agreed to the use of recorders and cameras. In the beginning of the interviews, consent was once again asked for and thus audio-recorded. It was ensured that interview transcripts and audio files were only accessed by the researchers and were not shared with anyone. Confidentiality and anonymity were ensured throughout the data gathering and analysis.

## Results

### Research participants

A total of 11 leprosy patients were recruited and interviewed, 7 males and 4 females. The patient’s age varied from 17 to 43 years. Interestingly, a third of the interviewees were migrant workers who became sick in India and were referred to Nepal for treatment.

Regarding the HCWs, a total of 15 participated in the study including physicians (n = 9), screening supervisors (n = 2), community-based rehabilitation workers (n = 1) and counselors (n = 3). The physicians interviewed were mainly dermatologists (n = 6) and generalists (n = 3), with different levels of experience ranging from junior residents to professors and clinical directors. The overall number of participants in each site is described in Table 1.

**Table 1:** Number of patients and HCWs interviewed by site.

Healthcare facility	Leprosy patients		Number of HCWs
	Male	Female	
**Koirala Institute of Health Sciences (BPKIHS)**	2		6
**Lalgadh Leprosy Hospital and Services Centre (LLHSC)**	4	2	4
**Anandaban Hospital and Patan clinic**	1	1	4
**Itahari PHC**		1	1

### Socio-professional dimension

Almost all of the patients were of low socio-economic status, living in houses made of mud walls with thatched, tin or bamboo roofs. Only two patients stated to have cemented houses. A majority had access to water (running water at home or communal tap) and electricity, but no toilets.

All HCWs recognized that leprosy affected mainly the poorest of the poor, living in austere conditions characterized by a lack of sanitation. They stated that this is an important factor that contributed to the spread of the diseases and is a challenge that must be addressed for disease control.

“Leprosy is a vicious disease. It affects very poor people with poor sanitation that live in very narrow conditions. In this problem of overcrowding it is difficult to educate because of the poor sanitation, the poor hygiene… We have to improve sanitation and economic conditions. We have to use a holistic approach economically socially and physically.” (LalH1)

Furthermore, all of the patients had a manual job (carpenters, construction workers and farmers), leading to difficulties for them due to complications of the disease.

“I have difficulties holding items. My wounds make it difficult to plough land.” (lalP2)

Some patients described that they lost their jobs due to the disease and are having difficulties finding new job opportunities causing great financial stress. One patient also described that when he was young, he was forced to abandon school when he became sick.

Most of the HCWs were aware that patients were confronted to social exclusion directly linked to disability and incapacity to work. To address the issue, they stated that leprosy centers also invested on socio-economic activities.

“We also work for economic rehabilitation- we are giving sometimes seed money, they will start a small business and try to earn money and they can start making economies and start their own business. That helps integrate them in the community and fight stigma.” (LalH2)

### Biomedical dimension

The most common manifestations of the disease the patients interviewed reported were skin patches (n = 11), loss of sensation of hands and feet (n = 6), loss of vision (n = 1), deformities (n = 3), ulcers (n = 3), infections (n = 2), foot drop (n = 2) and amputation of toes or feet (n = 2). Most of patients had to take a one-year course of treatment. Almost all patients reported a long time before seeking care due to a lack of knowledge of the disease. It is only when complications occurred (burns, wounds, ulcers or disfigurement) that they sought health care.

“I did not know it is serious. I got something like blisters from burns from fire. I did not give much attention to the wounds. My friend recommended me to come here. Now I have wounds as well as deformities.” (LalP1)

All of the patients interviewed claimed to take MDT, which is distributed for free in Nepal at PHC. The patients who were coming from neighboring India expressed they were motivated to come to Nepal in order to receive free treatment, and interestingly perceived the medication to be more effective than the one they received in India. However, most patients expressed having difficulties accessing care due to long distances and an important financial burden related to high travel costs and having to miss work.

“For 2–3 months I could not work, as I was weak. I could not go to get water (2 hours walk) and I did less household works. Also, it has been very difficult because I live far away and the expenses are high for transportation to come here (Lalgadh Hospital) “(Lal P4)

HCWs stated that many patients attended and were hospitalized to manage medicine related leprosy reactions, particularly in the referral centers. HCWs stressed the importance of counseling the patients regarding leprosy reactions that can develop even under treatment, as many patients may believe that the symptoms are due to the medication itself or that it is not effective against the disease which can lead to treatment discontinuation.

“For leprosy reactions, if there is no proper counseling they can think that it is a new infection. Sometimes they are fed up with the doctors, because they think that they have already taken Multidrug Therapy, that they are cured but they still have symptoms. So, they think the treatment we give is not effective.” (LalH3)

All of the HCWs explained that they were aware of the importance to take into account the overall health of the patient and avoid a disease centered approach. However, due to a heavy workload and lack of resources this is often difficult.

“The problem we have is salaries for staff. Because of the financial crisis we can’t increase the salaries of workers and the staff doesn’t have enough for their family.”(LalH3)

Some HCWs explained how they organized specific clinics and other mechanisms to give more time to evaluate the patient and facilitate referral to other specialists on a case to case basis. For example, in BPKIHS patients who are diagnosed with the disease are summoned to attend a leprosy clinic which is organized once a week in order to give more time for proper counseling. During this one-day leprosy clinic there is also an ophthalmology clinic organized for the specific evaluation of ocular complications of the disease.

On top of the individual counseling during consultations, both leprosy referral centers developed specific self-care training programs of one or two weeks, where patients are thoroughly taught about the disease, medication and preventive measures in order to prevent disabilities. Besides promoting self-care practices, these programs help patients cope with the disease and facilitate the transition of patients back into their communities. In order to attain these objectives, these programs rely on former leprosy affected patients referred to as facilitators, who demonstrate and practice with the patients these preventive measures. They also provide the much-needed psycho-social support and share their experiences explaining how they could adapt their daily life with the disease.

The use of formal counselors such as the facilitators also allows to address the problem of workload of physicians as it discharges them of a part of counseling activities. This was feasible for the referral hospitals but proven difficult for the other healthcare facilities evaluated.

“The problem is that we don't have a separate counselor. This is one part that I was trying to do for so many years”. BPKH3

Although very effective, these activities require significant financial and human resources investments by the hospitals.

### Cognitive dimension

#### Health beliefs

Although a majority of patients were aware that leprosy was an infectious disease, several patients still expressed different health beliefs even though they have been under treatment for several months. The oldest and most prevalent belief encountered was that the disease was a curse from god or sins from a previous life. Other patients associated the disease with heat, insects or even dirt in the blood.

“Leprosy I am not sure. Maybe it is a curse; it is due to any bad deeds in my previous life.” (LalP4)

“As I was working in Madras, where the temperature is very high. I also ate a lot of spicy food. I believe my disease is due to this, the heat and spicy food”. (LalP10)

All the HCWs also described how erroneous beliefs are widespread despite the numerous campaigns broadly broadcasted at the time of elimination (until 2010) and an important problem as they contributed to stigma. Informing about the disease, its origin and modes of transmission are described by HCW as key elements in the counseling process.

#### Knowledge and health seeking behavior

Most patients could not recognize the symptoms when affected which partly explained the delay in seeking care. It was often someone else noticing the problem who recommended where the individual should go, most of the time a family or community member who already had leprosy and was cured.

“It’s been almost 5 years. I did not know about my disease at that time. Only after it became unbearable I came here.” (BPKP11)

Patients who attended the self-care programs described above demonstrated a better understanding of the disease and its origins.

HCW also stated that the patients’ knowledge regarding the disease remains poor and insist on the need to adapt health education campaign to the level of education of the patients in order to be effective.

“The government does campaigns and articles in the papers but people don't read. So they need to do things at the grassroots level to be effective”. (AnaH3)

To address this issue, HCWs from specialized institutions explained how they created self-help groups in the communities, composed of former leprosy affected patients who were trained in the diagnosis and basic counseling on leprosy. In their communities, these groups contribute to improve the understanding of the disease and early detection.

“They have self-help groups in the community of patients affected by leprosy but also other disabled people. They will also do campaigns hoping to raise awareness about the disease. We are mobilizing these people now. They also are trained to recognize leprosy and when they suspect a leprosy case they send back to us”. (LalH3)

### Treatment

Despite prevalent health beliefs, all patients were convinced that leprosy is curable once under treatment, either because they personally know someone who was cured before or because doctors or health workers told them. All patients had a sound understanding of the importance of regularly taking their medicine, as the physicians had emphasized this.

“If I do not take the treatment, maybe I will be handicapped, have deformities, maybe death.” (lalP2)

HCWs described satisfactory patient compliance to the treatment in general. However, in a minority of cases, they reported inadequate patient compliance due to the length of the treatment, usually 1 year, but also due to poor knowledge of the disease or the medications side effects. Indeed, certain patients did not see the link between taking the drug and preventing complications and the transmission to others. Skin pigmentation due to clofozamine was often cited as an example of a side effect that might discourage patients from continuing treatment. According to a majority of HCW, the general population was aware of this side effect and patients feared to be identified due to the visible change in skin pigmentation and therefore might prefer not to take the medication.

“For some people, it is very difficult for them to have this dark skin. How to explain to the other people about this problem and they usually say they have a blood problem or this kind of thing. They do not say they have leprosy”. (AnaH5)

#### Self-care practices

One of the main preoccupations expressed by a majority of HCWs interviewed was the importance on counseling regarding self-care practices to prevent complications.

“We tell the patient that they are also their own doctors. They can also take care of themselves. They have a responsibility as well. If they don't take care of themselves properly, they will develop complications”. (LalH2)

Most patients interviewed reported having been counseled on self-care practices in order to prevent disability. Interviewees at the specialized leprosy institutions benefited from the self-care programs. After attending these programs, certain patients purposefully used gloves to protect hands and shoes while walking.

“I wear gloves while handling hot or sharp objects. I also wear my slippers or shoes while walking. I have to be extra careful while doing my work.” (ItaP12)

### Psycho-affective dimension

Most patients expressed how the disease generated a lot of anxiety and stress once diagnosed, but also during and after treatment. Patients still viewed the image of the leper as one with disfigurements and all expressed their fear of developing these visible signs of the disease.

This is a disease where you do not know what will happen. As it can damage my nerves, eyes, ears, so it is a bit stressful.” (LalP1)

The patients who were suffering from reactions of the disease expressed how painful and difficult it was to handle.

I wish not even my enemies to get this disease.” (LalP2)

However, the main problem all patients interviewed expressed was related to stigma and social exclusion sometimes heavier than the clinical manifestations of the disease itself. As a result, people affected by leprosy tended to hide their disease from others, often suffering in silence.

“When I got this disease, my family members were disgusted by me so I left my house.” (BirP7)

HCWs also widely recognized stigma as the main issue leprosy patients have to face. In the referral hospital, HCWs gave the example of several patients who stayed there after the treatment was completed, sometimes for several years, because they were rejected by their families and could not go back home, with nowhere else to go.

All HCWs stated that leprosy not only affected patients biological and physical needs, but also social, spiritual and psychological needs which required support and counseling.

“Everybody needs to show their love to these people. Without that we cannot do anything; that is the most important thing. Because they are already excluded from their communities, they are feeling very sad. If you give them education and counsel properly with love, they will listen and that can make them feel better. Many times, they are sad and scared and, in these cases, they don't listen properly. If you will counsel properly with love, they will listen.” (LalH1)

HCWs working in the referral centers organized group counseling sessions where patients were encouraged to discuss their issues and finding solutions together. Another strategy reported was working on patients’ erroneous beliefs and teaching patients on how to inform others about their disease. However, HCWs from non-specialized institutions had their own preconceptions and seldom investigated patients’ beliefs.

“The disease was associated with a curse from god very often. So, once we give the diagnosis, I don't test their knowledge. I explain directly what the disease is, an infection caused by bacteria, and explain that if you are getting the treatment you get cured. So, I'm not going to ask them because we know that the knowledge is not good. We have to emphasize more on how to change the beliefs.”(BPKH4)

The use of cured patients as facilitators in the counseling programs is also an effective strategy used in the referral hospitals to address the patients’ emotional distress. By sharing their experiences and challenges they had to overcome, they are role models for other patients which empowers them and helps them overcome their own fears. They are able to relate on a personal level and help patients find coping mechanisms.

“We trained cured patients to become facilitators. They were leprosy patients so they can give their example to others. They can say what type of problems they had to face in the communities and how they overcame them.”(LalH2).

HCWs also try to involve the patients’ family and sometimes community members in the educational process. At the community level, self-help groups were established and are usefully contributing to reducing stigma.

### Patient projects

When we questioned patients regarding their projects or hobbies, this was a concept that was not very familiar to them. Most patients were unable to answer this line of questioning, probably due to cultural differences. One patient stated he wished to get married, one was planning to build a house and another wanted to improve his current house. A majority of HCWs expressed that patients have difficulty projecting themselves into the future for various reasons such as financial constraints, precarious health or simply their own culture, reported to be focused on the “present”.

A summary of the overall results is presented in Table 2.

**Table 2:** Summary of patients and HCWs perspectives.

Dimension	Patients perspective	HCWs perspective
Socio-professional dimension	• Patients are mainly of low socio-economic status • Most of the patients present difficulties working due to complications of the disease. • Employment and schooling of leprosy affected patients may be compromised due to the disease.	• HCWs in referral centers develop socio-economic activities to improve the patients’ conditions and reintegration into society.
Biomedical dimension	• Many patients seek treatment late when complications of the disease and disabilities already have developed. • All patients are receiving anti-leprosy treatment • Patients express difficulties in accessing care related to distance to healthcare facilities and financial implications (transportation and loss of work)	• The majority of HCWs try to take into account the patients general health status in their approach • Mechanisms are in place in order to allow for proper evaluation of the patient such as specific clinics or referral pathways.
**Cognitive dimension**	• Most patients still present a multitude of alternate health beliefs (curse from god, heat, insects) • Most patients (except those from urban middle class) fail to recognize the early symptoms of the disease due to a lack of knowledge which leads to late health care seeking • Patients are compliant with their treatment and are convinced it will heal them. • The majority of patients adopt self-care behaviors when taught how to do so.	• Most HCWs acknowledge the patients’ alternate health beliefs, but do not always take them into account in their educational approach. • HCWs mentioned that awareness raising/Health education campaigns were not adapted to the population education level • HCWs observe that frequent side effects and duration of treatment constitute a barrier for treatment compliance • HCWs consider counseling on self-care practices as a priority in order to prevent disability.
Psycho-affective dimension	• Patients expressed suffering related to high levels of stigma and social exclusion Most patients considered the uncertain/variable evolution of the disease as a source of stress and anxiety	• HCWs recognize stigma as the main problem patients have to face. • In order to fight stigma, the majority of HCWs emphasize the importance of working on patients’ erroneous health beliefs. • HCWs also recommended other strategies to combat leprosy related stigma such as involving the patients’ families and community members in the educational process and the creation of self-help groups in the community. • To address patient suffering and stress, HCWs organize group counseling sessions to allow patients to share feelings and experiences • Former leprosy affected patients are employed as facilitators in counseling programs which allows to educate and empower the patients. •
Patient projects	• Patients presented difficulties addressing this line of questioning (culturally inappropriate?), evoking marriage and the improvement of housing conditions	• NA

## Discussion

To the authors’ knowledge, this is the first time such a study has been carried out using the model developed by d’Ivernois and Gagnayre [24], applied mainly in western countries and for non-communicable diseases. This model allows apprehending the patients’ educational needs, considering the disease’ complexity, to develop personalized strategies for patient education.

Overall, our interviews show that patient educational needs are globally aligned with those perceived by the HCWs. The main problem that emerged was the persistent lack of general knowledge of the disease by the patients, despite years of awareness efforts being carried out by health authorities and professionals in Nepal [31]. The persistent poor knowledge of the disease is explained by HCW as a result of ineffective campaigns, which are not adapted to the populations’ literacy level. Patients fail to recognize the symptoms of the disease and seek health care very late, after 15 years sometimes, allowing for transmission and preventable complications and painful disabilities to develop. Ignorance of symptoms by patients as a reason for not seeking health care has been also reported in other studies [32–34].

We found that many people still ignore the origins of the disease and view it as a curse from god among other health beliefs. This contributes to the stigmatization and social exclusion of patients which constitutes a major source of suffering, psychologically, socially and economically. This is consistent with the findings of other studies in Nepal [35–38] as well as other endemic countries [39,40]. Although HCWs were very much aware of the persistence of these different beliefs of the disease prevailing among the patients, they do not always take them into consideration in their educational approach. We also found that the majority of HCW have little formal training in patient education. This may also contribute to the problem. Indeed, the management of the different dimensions of patient education is complex and requires training [41–43]. Research has shown that training in patient education has a positive impact on health worker practice and patient adherence to treatment and self-care [44–47], crucial aspects of leprosy care.

Another challenge in educating patients many HCWs cited was the lack of time due to an important workload, consistent with other studies [48–50]. Given the limitations in terms of trained health workforce in Nepal and financial resources for that, the role of lay and peer counselors should be considered. Already largely involved in the management of leprosy patients within the specialized health centers with positive results, they constitute a valuable resource to correctly teach patients from the same origin with similar experience they had [14,51]. Interviewed patients themselves tend to confirm they could play a role and are willing to share their own experience of the disease.

In our patient population, all reported taking MDT. However, HCWs reported that sometimes compliance is still a problem that they have to face mainly due to poor knowledge and medication side effects. A study conducted in Nepal showed that the majority of non-compliant cases were people from poor economic class family background [35], which is the case of most leprosy affected patients in Nepal. HCWs from specialized referral institutions try to address this issue through community-based rehabilitation (CBR) activities. Findings show that adopting CBR principles and community development projects can stimulate improvements in living conditions, self-esteem and acceptance of people affected by leprosy into the community [52–54]. In Nepal, the government has started supporting CBR of leprosy patients since 2010, with a plan to initiate CBR in partnership with NGOs in all 75 districts of the country [54].

Since we selected patients through health facilities, all of them were accessing care. However, when listening to their itinerary, we note that access to adequate care was requiring substantial financial resources. Most patients were required to travel long distances, sacrifice their work and livelihood in order to access services.

The current study has several limitations. The qualitative nature of the study limits characterization of the strength of the relationships described here. Additionally, the small sample may limit generalizability. However, the variety of patients and health professionals included from different healthcare facilities is on the contrary a real asset that gives a broad picture of the situation in Nepal. There may have been some selection bias within the study population as most of patients and CHWs were from specialized Leprosy referral centers and as such are likely to possess above-average knowledge regarding the disease. Furthermore, there may be a researcher bias as one investigator involved in the analyses has worked in a patient education unit. Therefore, we cannot rule out that personal experience may have influenced the analysis in spite of careful adherence to the analytical process. Finally, we note a contextual bias due to the different culture between the researchers and study population. Even though the interviews were conducted with the aid of a translator, some cases may have been affected by language barriers and subtleties as well as the important number of local languages.

## Conclusion

The results show that there remains a lack of knowledge regarding the disease contributing to high levels of stigma despite years of efforts to raise awareness on the disease by the leprosy control program of Nepal. Different pedagogical approaches should be developed, evaluated and adapted in order to attain the desired educational objectives.

Capacity building of HCWs at community level, properly trained particularly in counselling could be part of the solution to disseminate more effectively key messages about leprosy to any patient. The use of dedicated counselors, in particular former leprosy affected patients, should be generalized beyond the specialized health-care centers.

## Supporting information

S1 File Patient interview guide. (DOCX)

S2 File Healthcare worker interview guide. (DOCX)
