# Supplementary material for: “If you will counsel properly with love, they will listen”: A qualitative analysis of leprosy affected patients’ educational needs and caregiver perceptions in Nepal
Source: PLoS One. 2019 Feb 6;14(2):e0210955. doi: 10.1371/journal.pone.0210955 (PMC6364891; doi:10.1371/journal.pone.0210955)
Supplement: S1 File — (DOCX) [file pone.0210955.s001.docx]

**S1: Patient interview guide**

1. **Biomedical dimension**

- How long have you been sick?
- What treatment do you take?
- Do you have any leprosy related complications?
- Do you have any other health problems?

1. **Cognitive dimension**

- In your opinion, how does the disease occur? (Explore the mode of transmission)
- What happens when you get the disease (Explore the clinical manifestations)
- Do you think it can be prevented (how can you protect others of the disease?). If yes then how? (Explore means of prevention)
- Do you think it can be treated?
- Do you take your medications?
- In your opinion, why do you take several drugs?
- At what moment do you take them? At what frequency?
- What difference do you see between your medications?
- Do you think they are effective?
- How do you manage your illness daily?
- What else do you do to treat yourself? (p. ex alternative medicine, foot care, etc.…). And why?
- How do you obtain information about your disease when you need it?
- In what situations do you seek the doctor?

1. **Health-seeking behavior**

- In case of a health problem, who would you refer to in the first contact? (Pharmacy, PHC, traditional healer, etc.…)
- Do you have any difficulties seeking health care? (transportation, financial etc.…)

1. **Socioprofessional dimension**

- What are your housing conditions? (explore type of house, water and electricity availability, overcrowding, etc.…)
- What do you do in life? (job, studies, etc.…)
- What impact does your disease have on your work/studies?
- What are your hobbies?
- Since you contracted the disease, have you changed your habits?
- Are there things that seem compromised by your illness? (marriage, future of the family, etc.…)

1. **Psycho-emotional dimension**

- Are there situations that particularly stress you?
- Do your loved ones know that you have this disease?
- Does your illness have an impact on your relationship with others? And at work / school? (stigma?).
- If yes, how do you cope with this?
- Do you think that you can be helped? If yes then how?
- Do you need more help? Do you have any suggestions to better manage your disease?
- Do you think that education about your disease would be useful to you? And to others?
