# Supplementary material for: “If you will counsel properly with love, they will listen”: A qualitative analysis of leprosy affected patients’ educational needs and caregiver perceptions in Nepal
Source: PLoS One. 2019 Feb 6;14(2):e0210955. doi: 10.1371/journal.pone.0210955 (PMC6364891; doi:10.1371/journal.pone.0210955)
Supplement: S2 File — (DOCX) [file pone.0210955.s002.docx]

**S2: Healthcare worker interview guide**

**General Questions:**

1. Name of establishment: ...........................................................................................
2. The person interviewed: .........................................................................................
3. Function in the institution (and for how long): ..........................................................
4. Training: ...................................................................................................................

**Education and counseling practices for leprosy affected patients:**

1. What are the existing educational activities and programs? (staff or materials/tools specifically dedicated for patient education and request program documentation if available).
2. What is the origin story (history) of these educational practices or programs (funders, partners, evolution, etc.…)?
3. In your opinion, is it a priority. If yes, why?
4. What problems are education and counselling supposed to address? p.e Treatment (medication and self-care), Prevention of complications, Stigma, professional life, living conditions, Hobbies/Sport, food habits).
5. Do you address patients’ representation/beliefs of the disease? (hot and cold model, curse, witchcraft, karma, etc.…)
6. Do you address coping strategies?
7. Do you include other people in the sessions? (Family / Surroundings). If yes, in which cases?
8. What time is dedicated to education and counselling (during the consultation)? And are the educational activities:

 Practiced at the same time as an assessment or care

 Separated from care (educational intervention alone)

 This depends

1. What is the frequency of the sessions? (Per week, per month, per year ...)
2. What is your incentive/motivation to perform education and counselling? (financial, ranking/post, status, etc..).
3. How were you selected for this job?
4. How have you been trained in counselling and education?
5. What do you see as weaknesses and strengths of the educational activities (or programs)?
6. What are the difficulties you are facing?
7. What suggestions do you have for improvement?
